# Trochlear Nerve Palsy: A Systematic Review of Etiologies and Diagnostic Insights

**DOI:** 10.3390/diagnostics15233082

**Published:** 2025-12-03

**Authors:** Areti Alexandrou, Nicholas Georgiou, George G. Botis, Ioannis Vezakis, George Triantafyllou, Eirini Christodoulaki, Harris Pishiaras, Alexandros Samolis, Nikiforos Christakos, Theodosis Kalamatianos, Ioannis Lamprianidis, Ioannis Kakkos, George K. Matsopoulos, George Tsakotos, Ourania Tzortzi, Maria Piagkou

**Affiliations:** 1 Department of Anatomy, School of Medicine, Faculty of Health Sciences, National and Kapodistrian University of Athens, 11527 Athens, Greece; aretealexandrou3@gmail.com (A.A.); nicholasgeo2003@gmail.com (N.G.); botis_g@biomed.ntua.gr (G.G.B.); georgerose406@gmail.com (G.T.); irichris@msn.com (E.C.); pishiarasch@outlook.com (H.P.); alexsamolis@me.com (A.S.); christakosnikiforos@gmail.com (N.C.); lamprianidisio@gmail.com (I.L.); gtsakotos@gmail.com (G.T.); raniatzortz2@gmail.com (O.T.); 2 Biomedical Engineering Laboratory, School of Electrical and Computer Engineering, National Technical University of Athens, 15772 Athens, Greece; ivezakis@biomed.ntua.gr (I.V.); ikakkos@biomed.ntua.gr (I.K.); gmatsopoulos@biomed.ntua.gr (G.K.M.); 3 “VARIANTIS” Research Laboratory, Department of Clinical Anatomy, Masovian Academy in Plock, 09402 Plock, Poland; 4 Department of Neurosurgery, Evangelismos Hospital, National and Kapodistrian University of Athens, 10676 Athens, Greece; tkalamatian@med.uoa.gr; 5 Department of Biomedical Engineering, University of West Attica, 12243 Athens, Greece

**Keywords:** trochlear nerve, fourth nerve, cranial nerve IV, superior oblique, diplopia, ocular motor cranial neuropathy, ischemic neuropathy, skull base surgery, iatrogenic injury

## Abstract

**Background/Objectives:** Trochlear nerve palsy (TNP) is a clinically significant neuro-ophthalmic disorder with a broad and heterogeneous etiologic spectrum. Due to the trochlear nerve (TN)’s long intracranial course and its proximity to key neurosurgical corridors, it is particularly susceptible to injury. This systematic review aimed to synthesize contemporary evidence on TNP etiologies and highlight diagnostic considerations. **Methods:** Following PRISMA 2020 guidelines (PROSPERO registration: CRD420251150614), we systematically searched PubMed through July 2025 for studies reporting TNP etiologies. Given substantial heterogeneity in study populations and methodologies, a qualitative synthesis was performed examining study characteristics, patient demographics, etiological distribution, and clinical outcomes. **Results:** Thirty-three studies (n = 5785) met the inclusion criteria. Reported etiologies clustered into seven categories: congenital, vascular/ischemic, infectious/inflammatory, structural, traumatic, iatrogenic, and idiopathic. Congenital cases frequently demonstrated absence of the TN or superior oblique hypoplasia. Microvascular ischemia predominated in older adults with vascular risk factors and typically exhibited spontaneous recovery. Structural lesions (e.g., tumors, aneurysms) and trauma were major acquired causes, often associated with more persistent deficits. Iatrogenic palsy predominantly followed skull base and petroclival procedures; most cases resolved, although lasting dysfunction occurred after complex or radiosurgical interventions. A proportion of cases remained idiopathic, and many resolved spontaneously. **Conclusions:** TNP displays a broad etiologic spectrum with distinct clinical profiles and prognostic trajectories. Accurate etiologic classifications supported by targeted neuroimaging and focused clinical evaluation are essential for optimizing management and informing neurosurgical decision-making.

## 1. Introduction

Cranial nerve palsies (CNPs) are a heterogeneous group of neurological disorders that affect the sensory, motor, or autonomic functions of the head and neck. In neuro-ophthalmology, palsies of the oculomotor (III), trochlear (IV), and abducens (VI) nerves are particularly important because they control ocular motility. Disruption of these pathways leads to ocular misalignment, impaired binocular coordination, and gaze-dependent diplopia, often resulting in compensatory head postures. Patients may also exhibit vestibular and ocular motor signs that complement bedside ocular alignment testing and help distinguish trochlear nerve palsy (TNP) from skew deviation or other mimics [1]. Persistent misalignment is associated with musculoskeletal strain, visual fatigue, and reduced quality of life [2]. Beyond symptoms, visual function testing demonstrates quantifiable impairment across multiple domains in CNPs [3]. Among isolated ocular motor nerve palsies (OMNPs), TNP is the least frequent, accounting for approximately 20% of cases [4,5]. Population-based studies estimate an annual incidence of TNP at 5.7 per 100,000 individuals [6]. In pediatric cohorts, however, TNP is often the most common form of OMNPs, comprising up to 36% of cases [7].

The trochlear nerve (TN) has several anatomical peculiarities that increase its vulnerability during neurosurgical procedures. It originates from the trochlear nucleus in the dorsal midbrain, inferior to the inferior colliculus, and is unique among cranial nerves (CNs) in that it completely decussates and exits dorsally. After traversing the ambient cistern between the posterior cerebral and superior cerebellar arteries, it courses along the tentorial edge before entering the orbit through the superior orbital fissure, remaining outside the annulus of Zinn [8,9,10]. This long, slender intracranial course predisposes the TN to traumatic, vascular, and iatrogenic injury, particularly during posterior fossa and skull base surgery [11]. Functionally, the TN innervates the contralateral superior oblique muscle, which produces intorsion, abduction, and depression in adduction—actions that are critical for binocular fusion in downgaze [12].

Trochlear nerve dysfunction (TND) results in hypertropia and excyclotorsion [13], producing vertical diplopia most pronounced during near work, such as reading or stair descent. Diagnostic evaluation typically combines clinical features with confirmatory testing, such as the Parks–Bielschowsky three-step test [14]. However, atypical presentations, bilateral involvement, and overlap with skew deviation, congenital cranial dysinnervation disorders (CCDDs), or ocular myasthenia gravis can complicate diagnosis [15,16]. Congenital cases may remain compensated for decades through fusional adaptation, delaying recognition. High-resolution magnetic resonance imaging (MRI) has dramatically improved differentiation between congenital and acquired forms of TNP by revealing trochlear nerve absence (TNA), superior oblique hypoplasia (SOH), or subtle brainstem lesions [17]. Nonetheless, the etiological spectrum remains broad [11], and a subset of cases remains idiopathic.

This systematic review provides a comprehensive, state-of-the-art synthesis of TNP etiologies, drawing on population-based studies, clinical series, and case reports published up to November 2025. By classifying congenital and acquired causes, we aim to refine diagnostic pathways, highlight prognostic distinctions, and support surgical planning to minimize postoperative cranial nerve morbidity.

## 2. Materials and Methods

This systematic review followed the Preferred Reporting Items for Systematic Reviews and Meta-Analyses (PRISMA) 2020 statement [18]. The PRISMA framework ensured a transparent, reproducible, and methodologically rigorous process for study identification, screening, eligibility assessment, and inclusion. The review protocol was developed a priori and guided all subsequent stages, including study selection, data extraction, and quality assessment.

### 2.1. Search Strategy

A comprehensive search of the PubMed and Embase databases was conducted through November 2025 to identify studies reporting on the etiology of TNP. Searches in both databases were performed using their respective Advanced Search Builders and were restricted to Title/Abstract fields. The PubMed search used the following Boolean expression: (“trochlear nerve” [Title/Abstract] OR “cranial nerve four” [Title/Abstract] OR “cranial nerve IV” [Title/Abstract] OR “CN IV” [Title/Abstract] OR “CNIV” [Title/Abstract]) AND (“palsy” [Title/Abstract] OR “palsies” [Title/Abstract] OR “paralysis” [Title/Abstract] OR “dysfunction” [Title/Abstract] OR “paresis” [Title/Abstract]) AND (“study” [Title/Abstract]). A conceptually equivalent query using Embase field tags (ti, ab) and Embase syntax was constructed to capture the same search terms. To further improve search sensitivity, conference abstracts in Embase were reviewed to determine whether they matched potentially relevant full-text publications in PubMed that shared similar authors, titles, abstracts, and PICOS elements. These were defined as PubMed Similar Articles. No language, publication status, or study design restrictions were imposed at the identification stage to maximize sensitivity. Reference lists of all included studies were manually screened to identify additional eligible records. This review was prospectively registered in the International Prospective Register of Systematic Reviews (PROSPERO; registration number CRD420251150614).

### 2.2. Eligibility Criteria

Following the initial database search, duplicate records and articles with non-English titles were removed before further evaluation. Systematic reviews were excluded to avoid duplication of data. Case reports and articles that could not be retrieved were also excluded. Additionally, studies primarily focused on therapeutic interventions, imaging modalities, or surgical/diagnostic techniques, as well as reports deemed clinically irrelevant to TNP etiology, were excluded. Conference abstracts were considered as part of the grey literature and were excluded from the qualitative synthesis due to limited data availability. However, PubMed-Similar-Articles corresponding to Embase Conference Abstracts—defined by the similarities above—were included if they also met the eligibility criteria.

### 2.3. Risk of Bias and Certainty of Evidence Assessment

A formal study-level risk of bias assessment for each study was conducted using the Risk of Bias in Non-randomized Studies of Interventions (ROBINS-I) tool, which evaluates seven domains of potential bias, including confounding, participant selection, exposure assessment and potential misclassification, missing data, outcome measurement, and selective reporting of results. The certainty of evidence for each association was appraised using the updated Grading of Recommendations, Assessment, Development, and Evaluations (GRADE) approach. In this framework, observational studies were initially rated as high certainty; however, certainty was downgraded for risk of bias, inconsistency (evaluated based on the similarity of point estimates, overlap of confidence intervals, and statistical tests such as I^2^), indirectness, imprecision, or publication bias, and could be upgraded in the presence of large effect sizes or dose–response relationships. High and moderate certainty of evidence suggests that the actual effect is likely or very likely to be close to the estimated effect. In contrast, low or very low certainty indicates limited confidence in the results. Two reviewers independently evaluated each study, and disagreements were resolved through consensus. Studies including both pediatric and adult populations, as well as unilateral, bilateral, congenital, and acquired forms of TNP, were eligible to allow comprehensive subgroup analyses and capture etiological variability.

### 2.4. Data Extraction and Analysis

Data extraction was performed independently by two reviewers, with discrepancies resolved through discussion and consensus. A standardized extraction form was employed to maintain consistency and minimize subjective bias across studies. For each survey, details regarding authors, year of publication, country of origin, affiliated institution, study design, sample size, and inclusion criteria were recorded. Patient demographics (age, sex, and relevant clinical characteristics) were extracted together with the number and percentage of TNP cases. Each case was classified by etiology. Pertinent clinical and neuroimaging findings, concomitant cranial nerve involvement, and patient outcomes (resolution or persistence of TNP) were also documented. This structured approach ensured consistent data collection and enabled a comprehensive synthesis of TNP etiologies, prevalence, and associated clinical features.

Regarding clinical outcome data, these were extracted as reported in the primary studies. When standardized definitions for key endpoints (e.g., “recovery”, “time-to-resolution”, or “persistent deficit”) were lacking, outcomes were classified qualitatively according to each study’s criteria. Follow-up durations and time-to-resolution, where available, were also recorded. Incidence estimates were summarized by etiology, subgroup, and study sample characteristics.

## 3. Results

**Figure 1:**
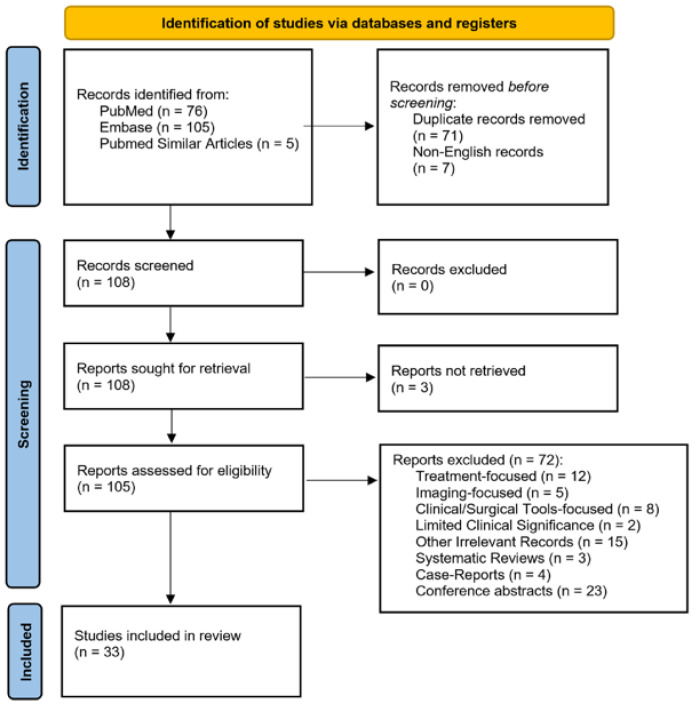
PRISMA 2020 flow diagram of study selection for systematic review. Records were identified through PubMed and Embase (n = 181). Seven non-English titles were excluded before screening. After screening 108 records and retrieving 105 full-text articles, 72 were excluded for reasons including treatment focus (n = 12), imaging focus (n = 5), clinical/surgical tools focus (n = 8), limited clinical significance (n = 2), irrelevant records (n = 15), systematic reviews (n = 3), case reports (n = 4), and conference abstracts (n = 23). Thirty-three studies were included in the final qualitative synthesis.

The initial search retrieved 76 articles indexed in PubMed, 105 in Embase, and five articles identified as Similar PubMed Articles covering the period from 1963 to November 2025. The PubMed Similar Articles were derived from relevant Embase conference abstracts and matched them based on similar authors, titles, abstracts, and PICOS elements. Following identification, 71 duplicate records and seven articles with non-English titles were removed. A total of 108 records were screened, and all were retrieved. Of these, three reports could not be retrieved, leaving 105 full-text articles to be assessed for eligibility. During title and abstract screening, duplicate systematic reviews were identified but retained for full-text evaluation to prevent duplicate data. Moreover, four case reports were identified but were also retained for assessment at the full-text stage in case the observations were notable for inclusion in the discussion section. At this stage, 72 studies were excluded for the following reasons: systematic reviews (n = 3), case reports (n = 4), treatment-focused (n = 12), imaging-focused (n = 5), clinical or surgical tools-focused (n = 8), limited clinical significance (n = 2), and other irrelevant topics (n = 15). Conference abstracts (n = 23) were considered part of the grey literature and excluded from the qualitative synthesis due to limited data availability. Ultimately, 33 studies met the eligibility criteria and were included in the qualitative synthesis. A detailed summary of study characteristics, reported etiologies, TNP frequency, and associated clinical findings is provided in Table 1. The selection process and reasons for exclusion are illustrated in the PRISMA 2020 flow diagram (Figure 1).

### 3.1. Classification of Trochlear Nerve Palsy (TNP) Etiologies

**Figure 2:**
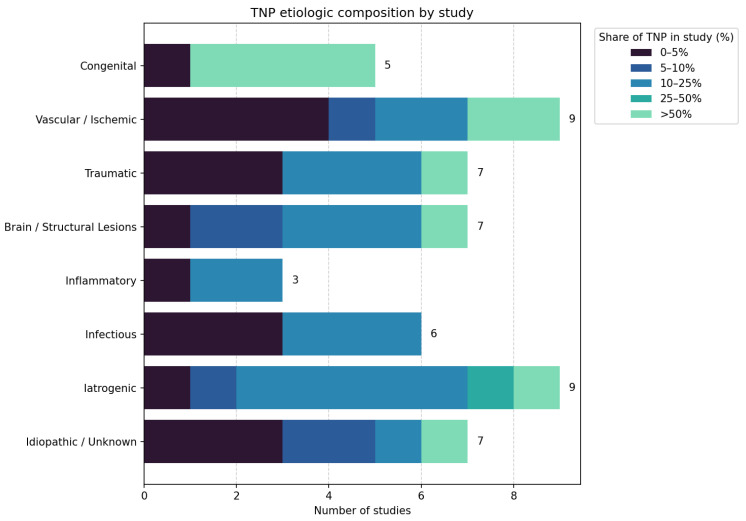
Summary of the etiologic composition of TNP across the included studies. Vascular/ischemic and traumatic causes were the most frequently reported, followed by structural and iatrogenic etiologies. Congenital TNP was more common in pediatric cohorts, while infectious and inflammatory causes were rarer. Idiopathic cases made up a small proportion (<10%) in most series. These patterns emphasize the heterogeneity of TNP and the impact of study population and design on the observed etiological distributions.

Based on data extraction and analysis (Table 2), the reported etiologies were classified into seven categories: congenital, vascular/ischemic, inflammatory/infectious, brain/structural lesions, traumatic, iatrogenic, and idiopathic (Figure 2).

#### 3.1.1. Congenital Trochlear Nerve Palsy (TNP)

Kim et al. reported 10 patients with congenital TNA, all of whom exhibited abnormal ocular motility [19]. Ellis et al. described nine patients with Brown syndrome, six of whom demonstrated TNA or TN hypoplasia (TNH), several associated with additional CCDDs, including Duane syndrome and congenital ptosis [20]. In a larger cohort, Yang et al. analyzed 97 cases of congenital superior oblique palsy and noted ipsilateral TNA in 73% of patients, frequently accompanied by SOH [21]. These findings were further corroborated in a retrospective review of 128 cases of unilateral superior oblique palsy (SOP), in which 88 patients demonstrated ipsilateral TNA [22].

#### 3.1.2. Vascular/Ischemic Palsy

Microvascular ischemia is consistently identified as the predominant acquired cause of TNP in older adults. In a cohort of 298 patients, Choi et al. reported microvascular ischemia in 23% of cases [23]. An extensive multicenter prospective study by Hörner et al., including 502 adults aged ≥ 50 years with isolated OMNPs, attributed 83.5% of cases to presumed microvascular ischemia, most often in association with vascular risk factors such as diabetes mellitus (DM), hypertension, hyperlipidemia, and smoking [24]. Choi et al. later demonstrated that among 82 patients with acquired TNP, 59.7% were linked to ischemia in the setting of systemic comorbidities, including hypertension, DM, coronary artery disease, microangiopathy, and dyslipidemia. Notably, 87.8% of these cases achieved complete recovery [5].

Earlier studies corroborate these findings: Berlit retrospectively analyzed 412 OMNPs and found that 52% of 25 isolated TNPs were attributed to DM or hypertension [25]; Keane documented 8 of 215 TNPs due to microvascular ischemia [26]; and Kumar observed that among 82 microvascular OMNPs, only 2% involved the TN, suggesting relatively lower susceptibility compared with other OMNPs [27]. More recently, Xue et al. reported 92 cases of diabetic TNP within a retrospective cohort of 609 patients, typically presenting unilaterally [28]. Bhargavi et al. reported 30 patients with trochlear nerve palsy, of whom 10 had vascular or ischemic causes [29]. Collectively, these findings confirm that microvascular ischemia is a leading cause of TNP in older adults, particularly in the presence of vascular risk factors, and is associated with a favorable prognosis and high rates of spontaneous recovery. Contemporary neuro-ophthalmology series similarly report ischemia as the leading cause of acquired IV palsy in adults, with characteristic minor vertical deviations and a favorable prognosis [23].

#### 3.1.3. Infectious and Inflammatory Etiologies

Infectious causes of TNP are relatively uncommon. In a retrospective review of 412 OMNPs, Berlit attributed 4% to infectious etiologies [25]. Keane, in an analysis of 215 TNPs, identified herpes zoster (n = 3), tuberculosis (n = 4), and acute bacterial meningitis (n = 4) as causative factors [26]. Other reports have described three cases of rhino-orbital-cerebral mucormycosis and 11 cases of cysticercosis, 10 of which developed bilateral palsies due to cyst compression of the caudal aqueduct [26]. Gupta et al. reported two TNPs among 18 HIV-negative patients with herpes zoster ophthalmicus [30]. In contrast, in a larger cohort of 330 patients with herpes zoster–related cranial neuropathies, Tsau et al. identified only one case involving the TN, associated with Ramsay Hunt syndrome [31]. Bhargavi et al. reported five patients with infectious causes of TNP, including septic cavernous sinus thrombosis [29].

Inflammatory disorders also contribute to TNP. Berlit noted that 8% of 25 isolated TNPs were associated with multiple sclerosis [25]. In a prospective multicenter series of 502 OMNPs, Hörner et al. reported that 32.7% of cases were inflammatory in origin, with TNP comprising 15% of these [24]. Common associations included autoimmune disorders such as multiple sclerosis, myasthenia gravis, Tolosa–Hunt syndrome, and rheumatoid arthritis, as well as viral infections including varicella-zoster virus (VZV) and respiratory syncytial virus (RSV). Rare presentations have been documented. Oda et al. described Rathke’s cleft cyst with local inflammatory reaction producing isolated TNP, which resolved after surgical removal [32]. Taken together, infectious and inflammatory causes—though less common than vascular or traumatic etiologies—represent clinically significant mechanisms of TNP. They should be considered, particularly in younger patients and in those with systemic autoimmune or infectious backgrounds, where early recognition may alter management and prognosis.

#### 3.1.4. Brain and Structural Lesions

Structural lesions are an uncommon but clinically significant cause of TNP. Gentry et al. identified seven primary trochlear schwannomas among 250 patients, 3 of whom had neurofibromatosis type 2 (NF2) and 1 with neurofibromatosis type 1 (NF1) [33]. Keane reported 14 tumor-related cases, often associated with additional cranial neuropathies due to compressive effects [26]. More recent studies highlight diverse structural pathologies. Oda et al. described a pituitary adenoma presenting with persistent TNP, visual field deficits, and endocrine dysfunction [32].

Vascular lesions have also been implicated: Koskela et al. reported two cases of TNP among 121 ruptured aneurysms—one following rupture of an anterior inferior cerebellar artery (AICA) branch during childbirth and another after basilar aneurysm clipping [34]. Peluso et al. identified a distal superior cerebellar artery (SCA) aneurysm causing TNP in a series of 2201 aneurysms [35]. Choi et al. emphasized that aneurysms and tumors accounted for 9.8% of TNP cases, with many showing incomplete recovery [5]. Similarly, Hörner et al. reported nine structural causes among 71 patients with TNP, including brainstem infarctions, hemorrhages, demyelinating plaques, and cavernous malformations [24]. Gadgil et al. observed 18 TNP in 182 patients following posterior fossa tumor resections [36]. Overall, structural lesions—including petroclival meningiomas, pituitary adenomas, and posterior circulation aneurysms—represent less frequent but essential causes of TNP. Unlike microvascular ischemic palsies, these cases are more likely to result in persistent deficits, yet they remain among the most surgically treatable etiologies.

#### 3.1.5. Traumatic Palsy

Trauma is a major acquired cause of TNP, though reported prevalence varies across studies. Keane identified trauma in 113 of 215 TNP cases, representing the predominant etiology in that cohort [26]. In an extensive population-based analysis of 2.6 million patients with traumatic brain injury (TBI), Heo et al. reported 1851 OMNPs, with TNP the most frequent subtype (37.7%) [37]. Similarly, Christoff observed trauma in 19 of 89 TNPs (21.3%) among 575 OMNPs [38]. Choi et al. found trauma responsible for 19.5% of cases [5], while Gurung et al. noted a comparable prevalence of 18.4% in patients with TBI and neuro-ophthalmic manifestations [39]. Other series reported lower rates. Ogun et al. identified three TNPs among 59 OMNPs, two trauma-related, making the TN the least frequently affected [40]. Berlit ascribed trauma to 12% of 25 isolated TNPs [25]. Ono et al. reported only one TNP (2.3%) among 44 post-traumatic patients [41]. Rajeshwari et al. reported two cases of TNP attributable to traumatic causes among a cohort of 110 patients with other ocular motor cranial neuropathies [42]. Taken together, trauma represents a significant cause of TNP, particularly at the population level where TBI is common. The variability in reported prevalence across studies likely reflects differences in cohort size, study design, and diagnostic criteria.

#### 3.1.6. Iatrogenic Etiologies

TNP is a recognized complication of skull base and petroclival surgery. Golshani et al. reported one transient TNP in a prospective series of 10 pediatric patients with craniopharyngioma treated with a modified frontotemporal orbitozygomatic craniotomy [43]. Keane documented 30 postoperative cases within his cohort [26]. In epilepsy surgery, Cohen-Gadol et al. observed transient diplopia due to TNP in 9 out of 47 patients, resolving within 3–6 months [44]. Inoue et al. described a single transient case among 27 patients undergoing microvascular decompression for trigeminal neuralgia [45]. In comparison, Bal et al. reported one transient TNP after a suboccipital transtentorial approach in a cohort of eight patients [46].

Radiosurgical and petroclival interventions also pose risk. Gerganov et al. documented TND in one of four patients treated with gamma-knife radiosurgery for petroclival meningioma [47]. Liao et al. reported three partially resolved TNPs among 18 patients undergoing pretemporal trans-Meckel’s cave transtentorial resection of large petroclival meningiomas [48]. Morisako et al. identified three non-transient cases among 23 Grade I petroclival meningiomas treated via a combined transpetrosal approach [49,50]. In a subsequent series, one of 10 patients undergoing a purely endoscopic subtemporal keyhole anterior transpetrosal approach developed non-transient TNP [51].

Collectively, these findings highlight the trochlear nerve’s vulnerability during skull base and petroclival interventions. While most postoperative cases are transient and resolved within months, complex approaches—particularly in the petroclival region—carry a measurable risk of persistent deficits. Careful intraoperative dissection and minimally invasive techniques may mitigate this risk.

#### 3.1.7. Idiopathic/Other Etiologies

A proportion of TNP cases remain idiopathic despite thorough investigation. Hörner et al. reported unknown causes in 28 out of 75 OMNPs, several of which involved the TN [24]. Choi et al. identified five idiopathic TNPs, most of which resolved spontaneously [5]. Kumar described one idiopathic case among three TNP patients [27], while Berlit found that 20% of 25 isolated TNPs were idiopathic [25]. Ogun et al. reported one to three idiopathic cases in their OMNP series [40], and Choi et al. reported 11 of 69 TNPs without an identifiable cause [23]. Collectively, these findings demonstrate that a subset of TNP cases remains unexplained despite a comprehensive workup. Many resolve spontaneously, suggesting transient or subtle mechanisms not captured by standard diagnostics. However, persistent idiopathic cases underscore the importance of long-term follow-up and careful consideration of occult causes.

**Table 2:** Summary of published studies on the causes of trochlear nerve palsy (TNP). The table outlines study features, sample sizes, patient demographics, inclusion criteria, and the number and percentage of TNP cases. Reported causes are classified as congenital, vascular/ischemic, inflammatory/infectious, brain and structural lesions (such as tumors, aneurysms, hemorrhages), traumatic, iatrogenic, and idiopathic. The studies were systematically classified by etiology and arranged chronologically from the earliest to the latest publication year. At the end of the table, studies reporting mixed etiologies (i.e., more than one etiology) were included. Relevant clinical findings, neuroimaging characteristics, and associated cranial nerve palsies (CNPs) are also summarized. Abbreviations: AICA—anterior inferior cerebellar artery; ATL—anterior temporal lobectomy; BA—basilar artery; CNPs—cranial nerve palsies; CSF—cerebrospinal fluid; DM—diabetes mellitus; eATPA—endoscopic anterior transpetrosal approach; F—female; HZV—herpes zoster virus; M—male; mAPCTPA—minimal anterior–posterior combined transpetrosal approach; mFTOZC—modified frontotemporal orbitozygomatic craniotomy; MVD—microvascular decompression; NF—neurofibromatosis; OMCNP—ocular motor cranial nerve palsy; PCM—petroclival meningioma; PII—petrous internal interval PTMCTA—pretemporal trans-Meckel’s cave transtentorial approach; SCA—superior cerebellar artery; SOP—superior oblique palsy; SOH—superior oblique hypoplasia; SOTTA—suboccipital transtentorial approach; SOM—superior oblique muscle; TNA—trochlear nerve absence; TN—trochlear nerve; TRN—Trigeminal Nerve; VBA—vertebrobasilar artery; After excluding non-diabetic TNPs from a total of 609 isolated TNPs. The extended table is included as Supplementary Materials. * after exclusion of non-diabetic TNPs from a total of 609 isolated TNPs. ** percentage of the total 609 TNPs; *** percentage of the 7 TNPs caused by the seven primary trochlear nerve tumors; **** Data from 2.606.600 trauma patients in IBM MarketScan Research Databases (2007–2016), TNT—trochlear nerve tumor.

Author Year Country	Sample (n)	Patient Demographics: Mean Age (Range in Years) Sex (M/F)	TNP Cases n (%)	TNP Etiology		Key Findings [TNP Cases/Sample]
Kim et al. [19] 2010, Korea	10	(4–47) 8 M/2 F	10/10 (100)	Congenital (4 studies)	SOH Ipsilateral TNA	10/10: limited depression +excessive elevation in adduction + Torticollis in early life, head-tilt sign
Ellis et al. [20] 2012, USA	9	(8–82) 4 M/5 F	6/9 (66.7)		TNA or underdeveloped TN Brown Syndrome	3/9: SOH Ipsilateral, 3/9: SOP Contralateral
Yang et al. [21] 2012, Korea	97	(0–76) 65 M/22 F	71/97 (73)		SOP Ipsilateral TNA	71/97: SOM Hypoplasia (variable degree)
Yang et al. [22] 2015, Korea	128	(0–63) 76 M/52 F	88/128 (70.4)		SOP Ipsilateral TNA	88/128: Significantly smaller SOM area and volume in the paretic compared to the normal side
Gupta et al. [30] 2011, India	18	(21–39) 13 M/5 F	2/18 (11.1)	Inflammatory/Infectious (2 studies)	HZV Infection	2/18: Disseminated Herpetic Lesions + Post-Herpetic Neuralgia + HIV-Negative
Tsau et al. [31] 2020, Taiwan	330	(55.0 ± 17.0) 155 M/175 F	1/330 (0.3)		HZV Reactivation	1/330: Ramsay–Hunt syndrome (III, V, VI, VII) + Complete Ophthalmoplegia
Kumar et al. [27] 2020, Saudi Arabia	92	(18–90) 65 M/27 F	3/92 (3.2)	Vascular/Ischemic (2 studies)	2/92: (2.2) DM Unknown: 1/92 (1.1) Binocular Diplopia due to OMCNP	1/3 Patients with TNP Initially Presented with VI Palsy + 12 Months after developing TNP
Xue et al. [28] 2025, China	92 *	(44–86) 67 M/25 F	92 */92 (15.1) **		Diabetic TNP	92/92: Unilateral TNP DM Duration is the only Significant Severity Factor
Gentry et al. [36] 1991, USA	6	(28–64) 5 M/1 F	5/6 (71.4) ***	Brain and Structural Lesions (4 studies)	7 Primary TNΤs	1/6: NF-1+ Unilateral TNP 1/6: NF-2+ Bilateral TNP
Peluso et al. [35] 2007, Netherlands	11	(44–70) 6 M/5 F	1/11 (9.1)		Partially thrombosed Distal SCA Aneurysm	1/11: TNP Almost Resolved after 12 Months
Koskela et al. [34] 2014, Finland	121	(20–84) 55 M/66 F	2/121 (1.7)		Ruptured Intracranial Aneurysm	1/121: TNP at birth from aSAH of the Distal Branch of AICA 1/121: TNP alongside VI Palsy After Clipping BA-AICA
Gadgil et al. [36] 2018, USA	182	(0–18), -	18/182 (9.8)		Posterior fossa tumor	18/182: Hypertropia due to TNP 12/180: Hypertropia patients had permanent deficits 8/180: Hypertropia due to skew deviation
Christoff et al. [38] 2015, USA	575	243 M/328 F	82/575 (14.2) Traumatic: 19/82 (23.2)	Traumatic (5 studies)	Neuro-Ophthalmology and Oculoplastic Patients	3/575: TNP+ Hydrocephalus
Heo et al. [37] 2023, ****	1851	<65 years 1067 M/784 F	697/1851 (37.7)		OMCNP	TNP is more frequent than other OMCNPs at age > 40
Gurung et al. [42] 2024, Nepal	377	(3–85) 271 M/106 F	7/377 (1.84)		Traumatic Brain Injury 5/377 (1.3) Skull fractures 2/377 (0.53) Contusions	Neuroimaging findings: 2/7: Contusions 5/7: Skull fractures
Ono et al. [41] 2024, Japan	44	(42.8 ± 24.2) 38 M/50 F	1/44 (2.3)		After a traffic accident	1/44: Binocular TNP
Rajeshwari et al. [45] 2025, India	110	(35.6 ± 18.5) 69 M/41 F	2/110 (0.02)		OMCNPs	38/110: Optic nerve dysfunction 17/110: Disc Edema 3/110: Retrobulbar neuritis
Cohen-Gadol et al. [44] 2003, USA	47	-	9/47 (19)	Iatrogenic (8 studies)	ATL for Seizures	9/47: Transient TNP-Resolution in 3–6 months
Golshani et al. [43] 2009, USA	10	(1.5–17) 6 M/4 F	1/10 (10)		mFTOZC Craniopharyngioma Resection	1/10: Transient TNP-Resolution in Follow-up
Gerganov et al. [47] 2014, Germany	4	(38–55) 4 M	1/4 (25)		TRN post-radiosurgery	1/4: TNP after Total Petroclival Meningioma resection with CSF leak
Liao et al. [48] 2018, Taiwan	18	(31–75) 6 M/12 F	3/18 (16.7)		PTMCTA for PCM Resection	3/18: TNP Partially Transient-Partial resolution after 18 Months of Follow-up
Inoue et al. [45] 2021, Japan	27	(43–88) 13 M/13 F	1/27 (3.7)		MVD for TRN due to VBA Compression	-
Morisako et al. [52] 2021, Japan	23	(37–74) 4 M/19 F	3/23 (13%)		mAPCTPA for PCM resection	-
Bal et al. [46] 2023, UK	8	(35–69) 3 M/5 F	1/8 (13.3)		SOTTA for PII Lesions	1/8: TNP-Ipsilateral and Transient- after Right Lateral Inferior Tentorium Meningioma resection
Morisako et al. [53] 2024, Japan	10	(28–76) 2 M/8 F	1/10 (10)		eATPA for removal of Petrous Apex Lesions	1/10: VI Palsy Aphasia, after Meckel’s Cave Meningioma resection
Berlit et al. [25] 1991, German	412	(14–82) 184 M/228 F	Total: 25/412 Isolated:6/412	Vascular/Ischemic: 14/25 (56%), Idiopathic: 5/25 (20%) Inflammatory/Infectious:3/25 (12%), Traumatic: 3/25 (12%)		OMCNPS Accompanied pain is less frequent in TNP than in other OMCNPs
Keane et al. [26] 1993, USA	215	(9–83) 153 M/62 F	215/215 (100)	Traumatic: 113/215 (52.6). Inflammatory/Infectious: 35/215(16.3). Iatrogenic: 30/215 (13.9). 16/35: Meningitis, 11/35: Cysticercosis, 4/35: Tolosa-Hunt Syndrome, 3/35: Mucormycosis (Cavernous Sinusitis), 1/35: Sarcoidosis. Brain & Structural lesions: 23/215 (10.7) 14/23: Tumors, 4/23: Brainstem Strokes, 3/23: Brainstem diseases, 2/23: Cavernous Aneurysms Vascular/Ischemic: 8/215 (3.7), Congenital: 3/215 (1.4), Other: 3/215 (1.4)		TNP 88/215: Right TNP, 86/215: Left TNP, 41/215: Bilateral TNP
Ogun et al. [40] 2019, Nigeria	59	(1–84) 28 M/31 F	3/59 (5.1)	Traumatic: 2/3 (66.6), Idiopathic: 1/3 (33.3)		OMCNPs
Choi et al. [23] 2019, South Korea	235	(9–88) 185 M/113 F	68/235 (29)	Vascular/Ischemic: 36/68 (52.9) Idiopathic: 11/68 (16.1) Traumatic: 10/68 (14.7) Inflammatory/Infectious: 8/68 (11.7) Brain & Structural Lesions: 3/68 (4.4)		Ιsolated OMCNPs Traumatic etiology is highest in TNP among the other OMCNPs
Hörner et al. [24] 2022, German	502	(16–92) 273 M/229 F	75/502 (15)	Idiopathic: 28/75 (37) Inflammatory/infectious: 18/75 (24) 5/18: Myasthenia Gravis, 3/18: Multiple Sclerosis, 2/18: Tolosa-Hunt, 2/18: Paraneoplastic, 2/18: VZV, 1/18: Rheumatoid arthritis, 1/18: Bacterial Rhinosinusitis, 1/18: Viral Infection Vascular/Ischemic: 15/75 (20) Diabetes Mellitus Brain & Structural lesions: 9/75 (12) 7/9: Brainstem Infarctions-1/9: BA aneurysm,1/9: Sphenoid meningioma, Other: 1/75 (1.3)		-
Oda et al. [32] 2023, Japan	30	(6–83) 15 M/15 F	2/30 (6.7)	Brain & Structural Lesions: 1/2 Pituitary Adenoma Inflammatory/Infectious: 1/2 Rathke Cleft Cyst		1/30: TNP from Pituitary Adenoma Compression +Hormonal dysfunction + Visual Deficit + III Palsy 1/30: TNP from Rathke Cleft Cyst Inflammation
Choi et al. [25] 2024, South Korea	82	(59 ± 11.1) 58 M/24 F	82/82 (100)	Vascular/Ischemic: 49/82 (59.7) Traumatic: 16/82 (19.5), Brain and Structural Lesions: 8/82 (9.8), Idiopathic: 5/82 (6.1), Other: 4/82 (4.9)		TNP 8/82: Brain & Structural lesion+TNP + Neurological symptoms
Bhargavi et al. [29] 2025, India	50	(20–75) 23 M/27 F	30/50(60)	Vascular/Ischemic: 10/30 (20), Idiopathic: Intracranial hypertension: 6/30 (12), Inflammatory/Infectious: 5/30 (10), Traumatic: 3/30 (6), Other: 6/30		Nuclear or intranuclear lesions of cranial nerves III, IV, VI 20/50: Diabetes + 4/50: Miller-Fisher + 4/50: Septic cavernous thrombosis + 2/50: Aneurysm + 2/50: IgG4 related + 2/50: Garcin syndrome + 2/50: Polyneuritis cranialis + 1/50: ANCA vasculitis + 1/50: Tolosa–Hunt syndrome

## 4. Discussion

TNP is a complex neuro-ophthalmic disorder with a heterogeneous etiological spectrum. This systematic review demonstrates that TNP may arise from congenital, vascular, infectious, inflammatory, structural, traumatic, iatrogenic, and idiopathic causes, often through overlapping mechanisms that necessitate comprehensive diagnostic evaluation. Due to heterogeneity in study designs and reporting standards, a formal meta-analysis was not feasible; therefore, a structured qualitative synthesis was undertaken.

**Congenital etiologies** predominate in pediatric and young adult populations, frequently presenting with early compensatory head tilt, superior oblique hypoplasia, and associations with congenital cranial dysinnervation disorders (CCDDs). Radiological evidence of TNA or TNH supports a developmental basis [18,19,20,21], while population-based data confirm underdiagnosis in strabismus cohorts due to long-standing fusional adaptation [4,23].

Among acquired causes, **vascular ischemia** remains the leading etiology in adults, particularly older individuals. It is strongly associated with diabetes mellitus (DM), hypertension, dyslipidemia, and coronary artery disease [22,23,24]. Most microvascular palsies are transient and self-limiting, though up to 10% may later prove to be structural on follow-up imaging [23]. Diabetic TNP appears more closely linked to disease chronicity than to acute glycemic status [27], emphasizing the importance of thorough neuroimaging in all presumed ischemic cases to exclude occult compressive pathology.

**Infectious and inflammatory mechanisms**, though less common, carry significant diagnostic and therapeutic implications, particularly in younger patients. Reported pathogens include herpes zoster, tuberculosis, bacterial meningitis, mucormycosis, and cysticercosis [5,25,28,29,52]. Autoimmune diseases such as multiple sclerosis, myasthenia gravis, rheumatoid arthritis, and Tolosa–Hunt syndrome have also been implicated [5,23]. Secondary inflammatory reactions, such as those arising from Rathke’s cleft cysts, further illustrate the diverse pathophysiological routes through which cranial neuropathies may develop. A delayed TND associated with iophendylate-induced arachnoiditis has also been reported [53], underscoring the need for a detailed clinical history and correlation of radiologic findings.

**Structural lesions**—including pituitary adenomas, petroclival meningiomas, gliomas, lymphomas, primary TN tumors (TNTs), and posterior circulation aneurysms—are less common but clinically significant causes of TNP [23,24,25,30,32,33,53]. Compared with ischemic palsies, structural etiologies more frequently result in incomplete recovery. Early detection and timely neurosurgical intervention are therefore essential to minimize morbidity, particularly when associated symptoms such as visual loss or multiple cranial neuropathies raise concern for mass effect or cavernous sinus involvement.

**Traumatic and iatrogenic palsies** represent additional major categories of acquired TNP. Population-level studies consistently identify trauma as a frequent cause of ocular motor cranial neuropathies, with TNP especially common after traumatic brain injury (TBI) [5,38,39]. The TN’s elongated intracranial course, dorsal emergence from the brainstem, and proximity to the tentorial edge predispose it to shearing forces during acceleration–deceleration injuries. While many traumatic palsies improve over time, recovery is less predictable than in microvascular cases, and persistent torsion or hypertropia is not uncommon.

**Iatrogenic palsy** underscores the TN’s vulnerability during skull-base, transpetrosal, and petroclival approaches. Although most postoperative deficits resolve within months [40,41,42,43,44], persistent dysfunction may occur after complex, multi-corridor skull base procedures or radiosurgery [45,46,47,48]. Preoperative planning and careful intraoperative handling of the tentorial edge are therefore critical to risk mitigation.

Finally, **idiopathic cases** persist despite comprehensive evaluation, comprising approximately 5–20% of cohorts [5,22,23,24,38]. Many idiopathic palsies resolve spontaneously, likely reflecting transient ischemic or inflammatory mechanisms below the threshold of routine imaging. Persistent cases, however, require long-term follow-up to identify delayed structural or inflammatory etiologies. When deviation stabilizes but remains symptomatic, surgical interventions—such as adjustable bilateral superior oblique tendon advancement—may offer meaningful improvement in select bilateral cases [47].

**Future Directions** Future research on TNP should prioritize large-scale, multicenter, prospective studies to clarify incidence, natural history, recovery trajectories, and long-term outcomes across etiologic subtypes. Standardized diagnostic criteria and reporting frameworks for ocular motor nerve (OMN) neuropathies are urgently needed to reduce heterogeneity and permit meaningful cross-study comparison.

Advances in neuroimaging—including high-resolution MRI, diffusion tensor imaging (DTI), and tractography—may enable earlier and more precise detection of TNA, TNH, or subtle compressive lesions, particularly in cases currently labeled as idiopathic or ischemic [54]. Artificial intelligence (AI) assisted interpretive tools hold promise for automating image-based detection, enhancing pattern recognition, and supporting prognostic modeling [55]. However, the current literature lacks standardized datasets, consistent methodology, and transparent reporting of bias [54,55,56].

Translational research into neurodegenerative and neuroprotective therapies may benefit patients with traumatic and ischemic palsies, which currently rely primarily on supportive care [56]. Equally important are well-designed studies evaluating strabismus surgery, minimally invasive skull base techniques, and postoperative rehabilitation, which remain underrepresented in the literature despite their relevance in refractory or iatrogenic cases [57]. Addressing these gaps will refine diagnostic algorithms, improve prognostic stratification, and support personalized care for TNP patients.

## 5. Strengths and Limitations

This systematic review offers the most comprehensive and up-to-date synthesis of TNP etiologies to date, encompassing 33 studies published through November 2025. Adherence to PRISMA 2020 guidelines and PROSPERO registration ensures methodological transparency and reproducibility. The inclusion of both congenital and acquired etiologies provides a unified framework applicable to neurology, ophthalmology, and neurosurgery. Additionally, the application of ROBINS-I and GRADE methodologies enhances methodological rigor, offering structured evaluation of risk of bias and evidence certainty. Nevertheless, several limitations must be acknowledged. A meta-analysis was deemed inappropriate due to substantial variability in study populations and methodologies. The absence of standardized denominators, combined with small sample sizes, rendered quantitative pooling statistically invalid. Consequently, a narrative synthesis provided a more accurate and contextually meaningful summary of the available evidence. Most included studies were retrospective and single-center, introducing potential selection and reporting biases. The lack of standardized diagnostic definitions likely contributed to inconsistencies in etiology classification. Additionally, limited or absent long-term follow-up restricted the ability to evaluate recovery trajectories. Finally, publication bias cannot be ruled out, as surgically treated or atypical cases tend to be reported more frequently.

## 6. Conclusions

TNP is a multifactorial neuro-ophthalmic disorder with diverse etiologies and variable outcomes. Congenital and ischemic forms predominate in pediatric and elderly populations, respectively, while trauma and surgical interventions account for a substantial portion of acquired cases. Structural, inflammatory, and idiopathic mechanisms, though less common, remain clinically significant. Prognosis varies markedly by etiology: microvascular palsies typically resolve spontaneously with favorable outcomes, whereas traumatic, neoplastic, and iatrogenic cases more often lead to persistent deficits. These findings highlight the necessity for comprehensive clinical evaluation, systematic neuroimaging, and individualized management strategies. For neurosurgeons and clinicians, early recognition, meticulous perioperative planning, and interdisciplinary follow-up are vital to optimizing recovery, minimizing morbidity, and preserving long-term visual function.

## Figures and Tables

**Table 1:** Etiology of Trochlear Nerve Palsy (TNP) across included studies, including mixed-etiology cases. The table summarizes the number of studies, total sample size, TNP cases (n and percentage), and key findings for each etiological category. Studies with mixed or non-exclusive etiologies are included; in such cases, TNP cases are counted for each reported etiology. Percentages represent the proportion of TNP cases within the total sample of included studies for each etiology. Key findings highlight the predominant causes, laterality, and notable clinical features for each category. TNA—trochlear nerve absence.

Etiology	No. of Studies	Total Sample (n)	TNP Cases	Key Findings/Notes
			n (%)	
Congenital	5	244	175 (71.7)	SOP/SOH predominates; mostly ipsilateral TNA
Vascular/Ischemic	9	537	221 (41.1)	DM-related, aneurysms, strokes; mostly unilateral; mixed studies
Traumatic	7	726	137 (18.9)	Mostly post-TBI, traffic accidents, and fractures; mixed studies
Brain/Structural Lesions	7	623	67 (10.8)	Tumors, parasellar/cranial lesions; some bilateral TNP; mixed studies
Infectious/Inflammatory	9	880	69 (7.8)	HZV, Tolosa-Hunt, meningitis, sinusitis; transient in some cases; mixed studies
Iatrogenic	9	197	50 (25.4)	Post-surgical, MVD, craniotomies; some transient; mixed studies
Idiopathic/Unknown	7	576	59 (10.2)	Rare and heterogeneous; idiopathic isolated OMCNP and cases with unknown causes; mixed studies.

## Data Availability

All data are available upon reasonable request to the corresponding author.

## Supplementary Materials

The following supporting information can be downloaded at: https://www.mdpi.com/article/10.3390/diagnostics15233082/s1, Table S1. Description and decision criteria for each domain in ROBINS-I. Table S2. GRADE assessment of the certainty of evidence for TNP outcomes.

## Abbreviations

TNP	Trochlear Nerve Palsy
TN	Trochlear Nerve
CN	Cranial Nerve
CNPs	Cranial Nerve Palsies
OMNPs	Ocular Motor Nerve Palsies
TND	Trochlear Nerve Dysfunction
TNA	Trochlear Nerve Absence
CCDDs	Congenital Cranial Dysinnervation Disorders
DM	Diabetes Mellitus
MRI	Magnetic Resonance Imaging
TBI	Traumatic Brain Injury
PRISMA	Preferred Reporting Items for Systematic Reviews and Meta-Analyses
AICA	anterior inferior cerebellar artery
CNs	cranial nerves
GRADE	Grading of Recommendations, Assessment, Development, and Evaluation
HIV	human immunodeficiency virus
JBI	Joanna Briggs Institute
OMN	ocular motor nerves
OMNPs	ocular motor nerve palsies
PROSPERO	International Prospective Register of Systematic Reviews
RSV	respiratory syncytial virus
SOH	superior oblique hypoplasia
TNH	trochlear nerve hypoplasia
VZV	varicella-zoster virus
