# Supplementary material for: Trochlear Nerve Palsy: A Systematic Review of Etiologies and Diagnostic Insights
Source: Diagnostics (Basel). 2025 Dec 3;15(23):3082. doi: 10.3390/diagnostics15233082 (PMC12691472; doi:10.3390/diagnostics15233082)
Supplement: Supplementary file 1 [file diagnostics-15-03082-s001.zip › diagnostics-3912605-supplementary.pdf]

Study	Year	Country	Sample (n)	TNP Cases	Selection Bias	Performance Bias	Detection Bias	Attrition Bias	Confounding	Exposure Bias	Reporting Bias	Overall ROB	Notes / Subgroups
Kim et al.	2010	Korea	10	10/10	Low	Low	Low	Low	Moderate	Low	Low	Moderate	Pediatric, Congenital, Unilateral
Ellis et al.	2012	USA	9	6/9	Moderate	Low	Low	Low	Moderate	Moderate	Low	Moderate	Mixed age, Possible TNA, Ipsilateral/Contralateral
Yang et al.	2012	Korea	97	71/97	Low	Low	Low	Low	Low	Low	Low	Low	Pediatric & Adult, Congenital SOP, Ipsilateral TNA
Yang et al.	2015	Korea	128	88/128	Low	Low	Low	Low	Low	Low	Low	Low	Pediatric & Adult, Congenital SOP
Gupta et al.	2011	India	18	2/18	Moderate	Low	Low	Low	Moderate	Low	Low	Moderate	Adult, HZV Infection
Tsau et al.	2020	Taiwan	330	1/330	Low	Low	Low	Low	Low	Low	Low	Low	Adult, HZV Reactivation
Miller et al.	2023	USA	1	1/1	High	Low	Low	Low	High	High	High	High	Adult, Iophendylate-Induced Polyneuropathy
Kuma	20	Saudi	92	3/92	Low	Low	Low	Low	Moderate	Low	Low	Moder	Adult, Vascular/Isch

r et al.	20	Arabia										ate	emic, DM-related
Xue et al.	2025	China	92	92/92	Low	Low	Low	Low	Low	Low	Low	Low	Adult, Diabetic TNP, Unilateral
Koskela et al.	2014	Finland	121	2/121	Low	Low	Low	Low	Moderate	Low	Low	Moderate	Adult, Aneurysm-related TNP
Peluso et al.	2007	Netherlands	11	1/11	Moderate	Low	Low	Low	Moderate	Low	Low	Moderate	Adult, Distal SCA Aneurysm
Gentry et al.	1991	USA	6	5/6	Moderate	Low	Low	Low	Moderate	Low	Low	Moderate	Adult, Primary TN Tumor
Christoff et al.	2015	USA	575	82/575	Low	Low	Low	Low	Low	Low	Low	Low	Mixed age, Diplopia
Heo et al.	2023	****	1851	697/1851	Low	Low	Low	Low	Low	Low	Low	Low	Adult, OMCNP-related TNP
Gurung et al.	2024	Nepal	377	7/377	Moderate	Low	Low	Low	Moderate	Moderate	Low	Moderate	Pediatric & Adult, Traumatic TBI
Ono et al.	2024	Japan	44	1/44	Moderate	Low	Low	Low	Moderate	Moderate	Low	Moderate	Adult, Traffic Accident, Binocular TNP
Cohen-Ga	2003	USA	47	9/47	Moder	Low	Low	Low	Moderate	Moderate	Low	Moder	Adult, Iatrogenic TNP, Transient

dol et al.					ate							ate	
Golshani et al.	2009	USA	10	1/10	Moderate	Low	Low	Low	Moderate	Moderate	Low	Moderate	Pediatric, Craniopharyngioma Resection, Transient
Gerganov et al.	2014	Germany	4	1/4	Moderate	Low	Low	Low	Moderate	Moderate	Low	Moderate	Adult, TRN post-radiosurgery
Liao et al.	2018	Taiwan	18	3/18	Moderate	Low	Low	Low	Moderate	Moderate	Low	Moderate	Adult, PCM Resection, Partially Transient
Inoue et al.	2021	Japan	27	1/27	Moderate	Low	Low	Low	Moderate	Moderate	Low	Moderate	Adult, MVD for TRN
Morisako et al.	2021	Japan	23	3/23	Moderate	Low	Low	Low	Moderate	Moderate	Low	Moderate	Adult, PCM Resection
Bal et al.	2023	UK	8	1/8	Moderate	Low	Low	Low	Moderate	Moderate	Low	Moderate	Adult, PII Lesions, Transient TNP
Morisako et al.	2024	Japan	10	1/10	Moderate	Low	Low	Low	Moderate	Moderate	Low	Moderate	Adult, Petrous Apex Lesions, VI Palsy
Berlit	19	Germany	412	25/412	Low	Low	Low	Low	Moderate	Low	Low	Moder	Adult, OMCNPs, Vascular/Idio

et al.	91											ate	pathic/Traumatic
Keane et al.	1993	USA	215	215/215	Low	Low	Low	Low	Moderate	Low	Low	Moderate	Adult, Trauma/Inflammatory/Iatrogenic/Congenital TNP
Ogun et al.	2019	Nigeria	59	3/59	Moderate	Low	Low	Low	Moderate	Moderate	Low	Moderate	Pediatric & Adult, Traumatic/Idiopathic TNP
Choi et al.	2019	South Korea	235	68/235	Low	Low	Low	Low	Low	Low	Low	Low	Mixed age, Isolated OMCNPs
Hörner et al.	2022	Germany	502	75/502	Low	Low	Low	Low	Moderate	Low	Low	Moderate	Adult, OMCNPs, Idiopathic/Inflammatory/Vascular
Oda et al.	2023	Japan	30	2/30	Moderate	Low	Low	Low	Moderate	Moderate	Low	Moderate	Pediatric & Adult, Pituitary/Parasellar Tumors
Choi et al.	2024	South Korea	82	82/82	Low	Low	Low	Low	Moderate	Low	Low	Moderate	Adult, Vascular/Traumatic/Idiopathic TNP
Bhargavi et al.	2025	India	50	30/50	Moderate	Low	Low	Low	Moderate	Moderate	Low	Moderate	Adult/Mixed etiologies & 20/50 with DM Several Rare Syndromic Cases (IgG4, Garcin, ANCA, Tolosa-Hunt syndrome)
Rajeshwa	20	India	110	2/110	Moder	Low	Low	Low	Moderate	Mod	Low	Moder	Adult, OMCNP cohort,2/110 Traumatic

ri et al.	25				ate					erate		ate	TNP ,others with optic nerve dysfunction or neuritis , very low TNP prevalence
Gadgil et al.	2018	USA	182	18/182	Moderate	Low	Low	Low	Moderate	Low	Low	Moderate	Pediatric ,Post–posterior fossa tumor resection, Brain/structural lesions Hypertropia due to TNP, partially permanent deficits

Table S1 Risk-of-bias assessment of included studies using ROBINS-I. Domains evaluated: Selection, Performance, Detection, Attrition, and Confounding,Exposure Bias,Reporting Bias . Overall ROB: Low, Moderate, or High. Notes indicate population (Pediatric/Adult), TNP laterality (Unilateral/Bilateral), and etiology (Congenital/Acquired). "Not reported" indicates missing information.

Outcome	Number of Studies	Sample Size	Risk of Bias	Inconsistency	Indirectness	Imprecision	Other Considerations	Overall Certainty (GRADE)	Notes / Subgroups
TNP incidence	33	5,550	Low to Moderate	Low	Direct	Precise	–	Moderate	Pediatric & Adult, All etiologies

Unilateral TNP	26	5,310	Low to Moderate	Low	Direct	Precise	–	High	Congenital & Acquired
Bilateral TNP	15	2,300	Moderate	Low	Direct	Imprecise	Small sample in some studies	Moderate	Adult, Acquired
Congenital TNP	6	250	Low	Low	Direct	Precise	–	High	Pediatric, Unilateral
Acquired TNP	21	5,342	Low to Moderate	Moderate	Direct	Imprecise	Etiology heterogeneity	Moderate	Adult, Traumatic/Infectious/Vascular/Iatrogenic
Vascular/Ischemic TNP	11	1,050	Moderate	Low	Direct	Precise	–	Moderate	Adult, Unilateral & Bilateral
Traumatic TNP	9	810	Moderate	Low	Direct	Imprecise	Small studies	Low	Pediatric & Adult, Mostly Unilateral
Iatrogenic TNP	7	352	Moderate to High	Low	Direct	Imprecise	Small sample	Low	Adult, Unilateral
Infectious/Inflammatory TNP	8	400	Moderate	Low	Direct	Imprecise	Heterogeneous etiology	Low	Adult, Unilateral
Diabetic TNP	1	92	Low	–	Direct	Precise	Single study	Moderate	Adult, Unilateral

Table S2 :GRADE assessment of the certainty of evidence for TNP outcomes. Certainty was evaluated across five domains: Risk of Bias, Inconsistency, Indirectness, Imprecision, and Other Considerations. Overall certainty is reported as High, Moderate, Low, or Very Low. Notes

indicate population characteristics (Pediatric/Adult), TNP laterality (Unilateral/Bilateral), and etiology (Congenital/Acquired).
